# Early prosthetic joint infections treated with debridement and implant retention: 38 primary hip arthroplasties prospectively recorded and followed for median 4 years

**DOI:** 10.3109/17453674.2012.678801

**Published:** 2012-06-04

**Authors:** Marianne Westberg, Bjarne Grøgaard, Finnur Snorrason

**Affiliations:** ^1^Department of Orthopaedics, Oslo University Hospital, Oslo; ^2^Department of Orthopaedics, Drammen Hospital, Drammen, Norway

## Abstract

**Background and purpose:**

Debridement and retention of the prosthesis is often attempted to treat early prosthetic joint infection (PJI). However, previous studies have found inconsistent results, with success rates ranging from 21% to 100%, and little has been written in the literature about hip function. We have therefore analyzed the clinical and functional outcome of early PJIs treated with this procedure.

**Patients and methods:**

38 patients with early PJI after primary hip arthroplasty who were treated with debridement and retention of the implant between 1998 and 2005 were studied prospectively, with a median follow-up time of 4 (0.8–10) years. Early infection was defined as that which occurred within 4 weeks of index arthroplasty. The primary outcome measure was infection control. Functional outcome was assessed with the Harris hip score.

**Results:**

27 of 38 patients were successfully treated, with no signs of infection or continued antibiotic treatment at the latest follow-up. Median Harris hip score was 86 (47–100) points. In 9 of the 11 patients for whom treatment failed, infection was successfully treated with 1-stage or 2-stage reimplantation or resection. Intraoperative cultures were positive in 36 hips, and the most frequently isolated organisms were Staphylococcus aureus and coagulase-negative staphylococci (CoNS). 15 infections were polymicrobial, and only 8 of them were successfully treated with debridement and retention of the implant.

**Interpretation:**

Our data suggest that debridement and retention of the prosthesis is a reasonable treatment option in early PJI after primary hip arthroplasty, with satisfactory functional results.

Prosthetic joint infection (PJI) occurs with an incidence of around 1% after primary hip surgery (NIH consensus conference 1995, Gaine et al. 2000, Phillips et al. 2006). The number of patients requiring a joint replacement is steadily increasing, and the absolute number of PJIs will be rising (NIH consensus conference 1995). Recent publications even suggest that the incidence of infection is increasing (Kurtz et al. 2008, Dale et al. 2009).

In early PJI, debridement with retention of the implant is an attractive treatment option. This procedure reduces morbidity, and also length of hospital stay and costs, compared to 1-stage or 2-stage revision arthroplasty (Fisman et al. 2001). The reported success rates of this procedure range from 21% to 100% (Drancourt et al. 1993, Tsukayama et al. 1996, Brandt et al. 1997, Crockarell et al. 1998, Zimmerli et al. 1998, Barberan et al. 2006, Choong et al. 2007, Van Kleunen et al. 2010). Different definitions of acute postoperative infection, with length varying from 4 weeks to 3 months, combined with heterogenous patient series, make comparison of these studies difficult.

Here we evaluated the clinical outcome of early PJI treated with debridement and retention of the implant in an 8-year prospective cohort.

## Patients and methods

All primary total hip replacements (THRs) at our center were prospectively recorded as part of a quality registration between January 1998 and December 2005. We studied the early PJIs in this THR cohort. PJI was classified as being early when symptoms presented less than 4 weeks after arthroplasty, according to Segawa and Tsukayama (Tsukayama et al. 1996). Infection was diagnosed clinically and was based on the CDC definition of deep incisional surgical site infection (Mangram et al. 1999). 8 biopsies and joint aspirates were taken perioperatively, and a minimum of 2 tissue specimens had to be positive before we regarded the organism isolated as being the infecting organism.

40 consecutive cases of early PJI were identified, 38 of which were treated with debridement and retention of components and were included for further analysis (Table 1). The 2 patients who were not included were treated with suppressive antibiotic therapy.

**Table 1.** Patient demographics in 38 patients treated for early PJI

Variable	Value
Age in years, median (range)	75 (32–89)
Male/female	15/23
BMI, median (range)	29 (19–49)
ASA score, median (range)	2 (1–3)
Indication for arthroplasty	
OA/AVN/FS	33/3/2
Procedure	
Cemented/uncemented/reverse hybrid THA	36/1/1

BMI: body mass index; ASA: American Society of Anesthesiologists; OA: osteoarthritis; AVN: avascular necrosis; FS: fracture sequelae.

When an infection was diagnosed, the microbiological agent(s), the type of treatment, the duration of antimicrobial treatment, and the number of days in hospital were registered. Total stay in hospital was defined as the cumulative duration of all admissions required to treat the infection.The final clinical visit was conducted in 2008.

Recruitment to the study was contingent upon obtaining informed consent from the patient. The study was approved by the Regional Committee for Ethics in Medical Research (1.2007.364) and the Norwegian Data Inspectorate.

### Treatment

We aimed for early surgical debridement, preferably within 4 weeks of the index operation. The median time until initial debridement was 20 (11–63) days, and 33 of the patients were operated within 4 weeks. 29 patients underwent 1 soft tissue debridement and 9 patients underwent a second debridement median 14 (9–30) days after the first revision. None of the prostheses were found to be loose and there were no discharging sinus tracts.

The standard index approach was transgluteal. At soft tissue revision, the surgical strategy was to excise the wound margins and to remove all debris and necrotic soft tissue, followed by pulsatile lavage with several liters of saline. Any modular component was changed. None of the patients had received antibiotics prior to culture, and antibiotics were withheld until the biopsies had been obtained. An empirical intravenous antimicrobial regimen, vancomycin in combination with a b-lactam, was started perioperatively and maintained until the definitive microbiological results were known. Organism-specific antibiotics were then given. The duration of antimicrobial therapy was chosen arbitrarily by the treating surgeons, based on clinical signs and laboratory tests.

### Evaluation of outcome

The primary outcome measure was the presence or absence of PJI at the latest clinical follow-up. A second soft tissue revision was not regarded as a failure. At the latest follow-up visit, the patients were clinically assessed, and blood tests were obtained, including erythrocyte sedimentation rate (ESR) and C-reactive protein (CRP). Postoperative and follow-up radiographs were evaluated for signs of loosening (radiolucent lines more than 2 mm wide, migration, and osteolysis) and subperiosteal bone growth. Functional outcome was assessed by the use of the Harris hip score with a maximum score of 100 points (representing no disability).

A successful outcome was defined as lack of clinical signs and symptoms of infection, a CRP level of < 10 mg/L, ESR the same as before the index operation, and absence of radiological signs of loosening. Failure was defined as relapse of infection, with isolation of the same or another pathogen.

### Statistics

Fisher’s exact test was used to compare categorical variables. A p-value of < 0.05 was considered significant.

## Results

### Treatment outcome (Figure)

After a median follow-up time of 4 (0.8–10) years, treatment with soft tissue debridement and retention of the prosthesis was successful in 27 of the 38 hips. These patients had no clinical signs of infection and no longer had antimicrobial therapy. In the patients treated within 3 weeks of the index operation, 18 of 24 healed, and 9 of the 14 infections that were treated after 3 weeks also healed (p = 0.7). Thus, treatment failed in 11 of the 38 patients.

**Figure:**
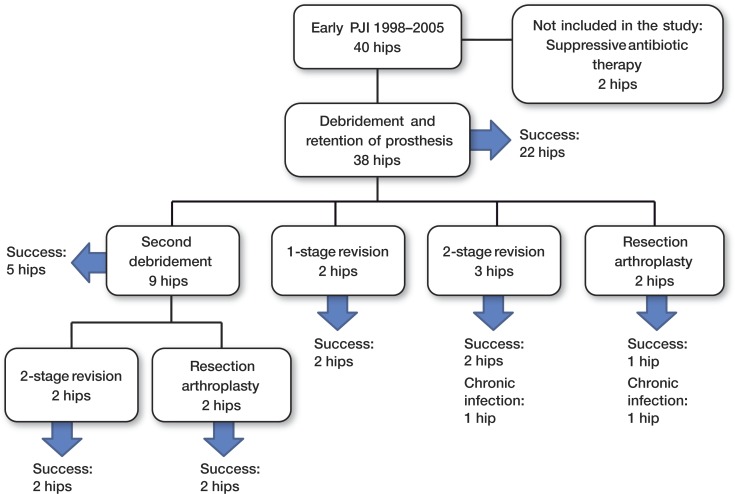
Flow chart of 38 hips with early PJI treated with debridement and implant retention

In the patients in whom treatment failed, soft tissue debridement was performed median 5 (4–7) weeks postoperatively. The failures occurred median 7 (5–133) weeks after the index operation. 6 of the failures were managed successfully with 1- or 2-stage revision arthroplasty. In 3 patients, the infection was controlled by removal of the prosthesis without reimplantation. In 1 patient, 2-stage exchange failed and the infection was treated with chronic antibiotic suppression. 1 patient had the prosthesis removed, but the infection was not controlled despite several attempts at additional soft tissue revisions. This patient was immunocompromised because of a kidney transplant.

### Functional outcome

26 of the 38 patients were completely rated with Harris hip score at the latest follow-up. Overall, median Harris hip score was 79 (25–100) points. In the group successfully treated with retained prosthesis and no signs of infection, median Harris hip score was 86 (47–100) points. In the patients who were reoperated with removal of the prosthesis, with or without reimplantation, median Harris hip score was 66 (25–90) points. The number of patients rated in these 2 groups were 20 and 6, respectively.

### Microbiology

Intraoperative cultures were positive in 36 of 38 patients. The most frequently isolated organism was *Staphylococcus aureus, *which was the sole causative pathogen in 10 hips. Altogether, it was isolated from 21 patients. None of the strains were methicillin-resistant. Coagulase-negative staphylococci (CoNS) were the sole causative pathogen in 7 hips and there were 18 isolates in total. 4 were methicillin-resistant. 15 of the infections were polymicrobial (Tables 2 and 3).

**Table 2.** Microbiological findings and treatment outcome in 38 patients treated for early PJI

A	B	C	D	E
Coagulase-negative staphylococci	7	4	3	
*Staphylococcus aureus*	10	10		
Streptococci	2	1	1	
Enterococci	1	1		
*Propionibacterium acnes*	1	1		
Culture-negative infection	2	2		
Polymicrobial infection	15	8	5	2

A Microorganism B No. ofepisodes C PJIs cured with debridement and retention of prosthesis D PJIs cured after 1- or 2-stage exchange or resection arthroplasty E Chronic infection

**Table 3.** Details of 15 polymicrobial PJIs treated with debridement and retention of the prosthesis

	Infecting organisms	Outcome after debridement and implant retention	Additional surgical procedures	Final outcome
1.	S. aureus + E. coli	Success		
2.	S. aureus + Streptococcus	Failure	2-stage exchange arthroplasty	Success
3.	CoNS + Enterococcus + Corynebacterium	Failure	2-stage exchange arthroplasty	Failure
4.	S. aureus + MRSE + Enterococcus	Success		
5.	CoNS + Streptococcus	Success		
6.	CoNS + MRSE	Failure	2-stage exchange arthroplasty	Success
7.	S. aureus + CoNS	Failure	2-stage exchange arthroplasty	Success
8.	S. aureus + CoNS	Failure	1-stage exchange arthroplasty	Success
9.	S. aureus + CoNS	Failure	Resection arthroplasty	Success
10.	CoNS + Enterococcus	Success		
11.	S. aureus + Enterococcus	Success		
12.	S. aureus + E. coli	Success		
13.	S. aureus + Streptococcus	Failure	Resection arthroplasty	Failure
14.	S. aureus + CoNS + Enterococcus	Success		
15.	S. aureus + MRSE	Success		

CoNS: coagulase-negative staphylococci; MRSE: methicillin-resistant staphylococcus epidermidis.

All 10 monomicrobial S. aureus infections were successfully treated with debridement and retention of the prosthesis. Only 8 of the 15 polymicrobial infections were treated successfully, as compared to 19 of the other 23 PJIs (p = 0.07). 4 of 7 monomicrobial CoNS infections, 1 of which was methicillin-resistant, were debrided with successful outcome (Table 2).

### Antimicrobial therapy

When definitive microbiological results were known, b-lactam drugs (cloxacillin, dicloxacillin, and penicillin)—alone or in combination with clindamycin—were used in 30 of the 38 hips. Subsequently, 8 hips were treated with linezolid and 3 hips were treated with a rifampicin combination. The median total duration of antimicrobial therapy, including treatment when additional revision procedures were performed, was 7 (3–39) weeks. The median duration of antimicrobial therapy for the 27 patients who were treated successfully was 7 (3–27) weeks. The median total stay in hospital was 6 (2–14) weeks.

### Complications and reoperations

2 patients developed nephrotoxicity, which was vancomycin-induced in 1 patient and b-lactam-induced in one with a kidney transplant. 10 patients died during the follow-up. All but 1 death had causes unrelated to the infection. 8 patients had dislocation of the hip postoperatively.

## Discussion

Our main finding was favorable outcome after debridement and retention of the prosthesis in early PJIs after primary THR. Patients who were treated successfully had a satisfactory functional outcome with a median Harris hip score of 86 points at the latest follow-up. Moreover, we found a high rate of polymicrobial infections with a tendency of poorer outcome.

One of the patients was immunocompromised because of a kidney transplant. This patient developed a severe polymicrobial PJI, failed all treatment efforts, and eventually died of the infection. This patient was quite different from the otherwise homogenous patient group, with an expected poorer outcome. If we exclude this patient, the treatment would have been successful in 27 of 37 patients. It would not have affected our general findings, though.

The recommended length of time between arthroplasty, or signs of infection, and soft tissue debridement is an important and much debated question. Long duration of symptoms has been found to increase the risk of treatment failure, and hence soft tissue debridement within a week after debut of symptoms is recommended (Brandt et al. 1997, Tattevin et al. 1999, Marculescu et al. 2006). Others recommend this treatment within 3 weeks after arthroplasty (Zimmerli et al. 2004). We do not have data on duration of symptoms, but we did not find a time-dependent association between treatment failure and time from arthroplasty. This could be because all the PJIs were treated within a relatively short time after arthroplasty, or it could be due to the low number of patients.

The most common microorganisms cultured in PJI are *Staphylococcus aureus* and CoNS, which account for more than half of the cultures (Tsukayama et al. 1996, Pandey et al. 2000, Widmer 2001). CoNS and *S. aureus* were the most frequently isolated organisms in the present study also. CoNS were present in 10 of 15 polymicrobial infections, and in all 18 CoNS isolates were detected. This demonstrates how important CoNS are in acute PJI, and not only (as traditionally considered) in late infections.

The number of methicillin-resistant CoNS (MRSE) infections in our study was low, which contrasts with other PJI reports on increasing numbers of MRSE infections (Stefánsdóttir et al. 2009). We have no clear explanation for the low incidence of MRSE, but it may have been due to the small material. In addition, our study involved only acute infections, and methicillin resistance is increasing with the number of reoperations and duration of infection. The phenotyping methods used to test CoNS for methicillin resistance in the period 1998–2005 were also less sensitive than the molecular methods used today.

*S. aureus* has been associated with higher failure rates (Brandt et al. 1997), but this correlation has not been shown in other series (Zimmerli et al. 1998, Barberan et al. 2006). We did not find this tendency in our material. None of the *S. aureus* strains in our series were methicillin-resistent.

We found that 15 of 38 infections were polymicrobial. This is somewhat higher than previously reported in the literature, where polymicrobial PJI has accounted for 5–22% of all PJI (Tsukayama et al. 1996, Gardner et al. 2011, Van Kleunen et al. 2010). In 2 recent studies, polymicrobial PJIs were more frequent in the early infections (Moran et al. 2007, Marculescu and Cantey 2008). The fact that our series only involved early PJIs may explain the high number of polymicrobial infections. Polymicrobial PJI has been associated with poor outcome (Jackson and Schmalzried 2000, Marculescu and Cantey 2008). We also found lower success rates with the polymicrobial infections than with other infections, but this was not statistically significant (p = 0.07). This supports previous reports about polymicrobial PJIs being a prognostic factor for failure. The polymicrobial infections in our series also had a higher frequency of enterococci and Gram-negative organisms than in the monomicrobial PJIs. These organisms are known to be difficult to treat, and may partly explain the inferior treatment results in the polymicrobial PJIs.

The optimal length of antimicrobial treatment of PJI is not clear. Current recommendations are 3 months in patients with early PJI of the hip (Zimmerli et al. 1998). It has recently been suggested that treatment may be restricted to 6 weeks (Bernard et al. 2010). The antimicrobial therapy in our material was not standardized, with great time variation, but the median time of treatment was considerably shorter than the recommended 3 months. This may support the suggestion of shorter duration of antimicrobial therapy in early PJI.

A previous report has suggested that attempting salvage of the prosthesis with soft tissue debridement may reduce the chance of successful reimplantation later on (Gardner et al. 2011). In a review of the literature, Jackson and Schmalzried (2000) reported a success rate of 83% in 1,299 PJIs treated with 1-stage exchange. Historically, 2-stage exchange arthroplasty has been the treatment of choice in PJI, with success rates of around 90% (Tsukayama et al. 1996, Hsieh et al. 2004, Sanchez-Sotelo et al. 2009). Our findings are in accordance with these reports: an initial attempt at debridement does not lead to inferior results at later revision arthroplasty.

Data on functional results following debridement and retention of prosthesis are often lacking in the literature. Hence, the expected level of Harris hip score is not clear. Tsukayama et al. (1996) evaluated 28 patients treated with soft tissue revision for early PJI, and reported 70 points for Harris hip score on average. The functional results after 1-stage and 2-stage revision surgery have been reported, with Harris hip score between 69 and 87 (Tsukayama et al. 1996, Hsieh et al. 2004, Cabrita et al. 2007). In our series, the average Harris hip score in patients who were managed successfully with soft tissue revision was 86. This is not as good as anticipated after primary THR, but appears to be similar to other reported results after PJIs.

The present study had several limitations. First, the debridement procedure was not done using a standardized protocol, and individual surgeons may have performed the procedure differently. However, only 5 different surgeons were involved in the treatment of the infected THRs. Secondly, there were no established guidelines for the type and duration of the antimicrobial treatment; each regimen was chosen individually by the treating surgeon. The treatment changed during the study period. In the second half of the period, linezolid- and rifampin-containing regimens were used in some of the patients. Variations in choice of treatment may have influenced the treatment results. Finally, the study population was relatively small. It is therefore difficult to determine potential risk factors associated with treatment failure. The study was, however, strengthened by its prospective registration and homogenous study population.

In summary, our findings support the use of debridement and retention of the prosthesis in early PJI of the hip. The functional outcome after this surgical strategy appears to be acceptable. CoNS and polymicrobial infections are frequently the cause of early PJI, which emphasizes the importance of broad-spectrum antimicrobial treatment initially. It is necessary to have a high index of suspicion of PJI, and to respond without delay in order to optimize the treatment results with this surgical modality.
